# Advancing Safe Medication Administration Training for Direct Care Workers: Lessons and Next Steps Following a Pilot Program

**DOI:** 10.51894/001c.164412

**Published:** 2026-08-01

**Authors:** Madeline Moreno, Abigail Stevens, Bethany Duyser

**Affiliations:** 1 College of Osteopathic Medicine, Michigan State University

## 53

### Introduction

Medication administration in assisted living and adult foster care (AFC) settings is increasingly performed by direct care workers (DCWs), yet standardized training practices and competency expectations remain inconsistent. As medication regimens become more complex, insufficient preparation of direct care workers tasked with administering medications may increase risk for preventable medication errors.

### Objectives

This work evaluates the impact of a targeted educational intervention on DCW medication administration knowledge, confidence, and recognition of unsafe practices, and to inform development of a scalable training model.

### Methods

A pilot educational intervention was implemented across four adult foster care homes with 33 participating DCWs. Using a pre-post design, participants completed structured assessments evaluating previous training experience, proper medication administration knowledge, confidence in administration, and identification of unsafe medication practices before and after training. The curriculum emphasized error prevention strategies, and practical application within AFC settings.

### Results

Following the pilot program, participants demonstrated measurable improvements in medication knowledge and increased confidence in identifying unsafe administration behaviors. The effectiveness of the training was demonstrated through statistically significant improvements in knowledge scores (*p* < 0.05 across all sites) and self-reported improvements in confidence upon completion of the intervention.

### Discussion

This pilot study supports the feasibility and potential effectiveness of structured medication administration training for DCWs. As long-term care settings increasingly rely on non-licensed caregivers, standardized and accessible training programs are essential. Integrating practical medication administration instructions with foundational pharmacology and toxicology education may help DCWs develop the critical thinking skills necessary to recognize unsafe practices and prevent harm. Future directions include development of a longitudinal study to evaluate skill retention and medication error outcomes over time, refinement of digestible training materials tailored to the DCW scope of practice, and assessment of scalability across diverse care settings.

